# Transcriptomic Analysis of the Endangered Neritid Species *Clithon retropictus*: De Novo Assembly, Functional Annotation, and Marker Discovery

**DOI:** 10.3390/genes7070035

**Published:** 2016-07-22

**Authors:** So Young Park, Bharat Bhusan Patnaik, Se Won Kang, Hee-Ju Hwang, Jong Min Chung, Dae Kwon Song, Min Kyu Sang, Hongray Howrelia Patnaik, Jae Bong Lee, Mi Young Noh, Changmu Kim, Soonok Kim, Hong Seog Park, Jun Sang Lee, Yeon Soo Han, Yong Seok Lee

**Affiliations:** 1 Department of Life Science and Biotechnology, College of Natural Sciences, Soonchunhyang University, 22 Soonchunghyangro, Shinchang-myeon, Asan, Chungcheongnam-do 31538, Korea; cindysory@naver.com (S.Y.P.); bioksw@naver.com (S.W.K.); hwamux@naver.com (H.-J.H.); jong6922@daum.net (J.M.C.); elegangce@naver.com (D.K.S.); gabriermingu@naver.com (M.K.S.); hhowrelia.patnaik@gmail.com (H.H.P.); 2 Trident School of Biotech Sciences, Trident Academy of Creative Technology (TACT), Chandaka Industrial Estate, Chandrasekharpur, Bhubaneswar, Odisha 751024, India; drbharatbhusan4@gmail.com; 3 Korea Zoonosis Research Institute (KOZRI), Chonbuk National University, 820-120 Hana-ro, Iksan, Jeollabuk-do 54528, Korea; jblee@jbnu.ac.kr; 4 College of Agriculture and Life Science, Chonnam National University, 77 Yongbong-ro, Buk-gu, Gwangju 61186, Korea; annemi@hanmail.net (M.Y.N.); hanys@chonnam.ac.kr (Y.S.H.); 5 National Institute of Biological Resources, 42 Hwangyeong-ro, Seo-gu, Incheon 22689, Korea; snubull@korea.kr (C.K.); sokim90@korea.kr (S.K.); 6 Research Institute, GnC BIO Co., LTD., 621-6 Banseok-dong, Yuseong-gu, Daejeon 34069, Korea; 5022daniel@gmail.com; 7 Institute of Environmental Research, Kangwon National University, 1 Kangwondaehak-gil, Chuncheon-si, Gangwon-do 243341, Korea; sljun@kangwon.ac.kr

**Keywords:** *Clithon retropictus*, transcriptome, de novo assembly, functional annotation, simple sequence repeats

## Abstract

An aquatic gastropod belonging to the family Neritidae, *Clithon retropictus* is listed as an endangered class II species in South Korea. The lack of information on its genomic background limits the ability to obtain functional data resources and inhibits informed conservation planning for this species. In the present study, the transcriptomic sequencing and de novo assembly of *C. retropictus* generated a total of 241,696,750 high-quality reads. These assembled to 282,838 unigenes with mean and N50 lengths of 736.9 and 1201 base pairs, respectively. Of these, 125,616 unigenes were subjected to annotation analysis with known proteins in Protostome DB, COG, GO, and KEGG protein databases (BLASTX; E ≤ 0.00001) and with known nucleotides in the Unigene database (BLASTN; E ≤ 0.00001). The GO analysis indicated that cellular process, cell, and catalytic activity are the predominant GO terms in the biological process, cellular component, and molecular function categories, respectively. In addition, 2093 unigenes were distributed in 107 different KEGG pathways. Furthermore, 49,280 simple sequence repeats were identified in the unigenes (>1 kilobase sequences). This is the first report on the identification of transcriptomic and microsatellite resources for *C. retropictus*, which opens up the possibility of exploring traits related to the adaptation and acclimatization of this species.

## 1. Introduction

Mollusca comprises a highly diversified group of animals, with members distributed in almost every ecosystem. The molluscan habitats include terrestrial mountaintops and the hot vents and cold seeps of the deep sea. These creatures have adapted to terrestrial, marine, and freshwater habitats and constitute the second most speciose phylum of all animals. Despite their ubiquity across the globe, many thousands of molluscan species still remain undescribed. Among the known molluscs, approximately 80% are gastropods (snails and slugs, limpets, and sea hares). Gastropods have successfully occupied all marine and freshwater habitats and are the only group of molluscs to have invaded the land. They have been the subject of paleobiological and numerous other ecological, evolutionary, physiological, and behavioural studies [1,2]. Their undisputed variety of morphology, structure, and habits, augmented by their utility to humans throughout history as a source of food, ornaments, tools, and even pets, has made the members of the gastropod community valuable for research investigations [3,4].

Within the molluscan clades, Neritospina contains several families that have marine, freshwater, and terrestrial members. Neritidae, which comprises the largest family, includes marine, brackish, and freshwater lineages. The members of this family are among the most abundant intertidal molluscs on tropical and subtropical coasts. Most species live on rocky shores and coral reefs, taking shelter in crevices or under rocks or seaweed. Their herbivorous habit allows grazing on the thin layer of algae on rock surfaces. In estuaries, members of this family live on rocks, wood, and mangrove roots. Among Neritids, the genus *Nerita* is most closely associated with the marine environment, whereas species belonging to genera *Neritina* and *Clithon* preferably inhabit brackish or freshwater habitats. Among the identified species within the genus *Clithon*, *C. retropictus* (Martens, 1878) inhabits saltwater or freshwater and is among the most long-lived gastropods, with a lifespan of 12 years. This species has been reported in Japan, South Korea, Thailand, Southern China, and Taiwan, especially occurring on hard substrates, such as stones and concrete blocks suggesting the association of snail occurrence with stone presence [5,6,7,8].

In South Korea, this neritid species has been reported to exist in Jeollanam-do (South Jeolla Province), Gyeongsangnam-do (South Gyeongsang Province), and Jeju-do Island [9,10,11,12]. The details of this species, including shell morphology and ecology, were first determined from studies on the south coast of Jeju Island, after which it was identified from under rocks adjacent to a dry river bed on a muddy substrate along the north coast [7,12]. *C. retropictus* was also identified from a shell drift at Yaerae-dong, on the southern coast of Jeju Island and from a small, shallow brackish-water estuary southwest of Gangjeong Town. The population of this species (distributed in restricted habitats in South Korea) is continuously under threat due to changes in natural habitats, blocking of seawater due to dam construction, pollution, pathogens, and so on [13]. This species has been designated as an endangered class II species by the National Institute of Biological Resources, South Korea and is protected under law. To protect the valuable genetic resources of this species and to avoid its extinction, a high-throughput genome-scale identification of transcripts is necessary. Transcriptomic analysis is directly relevant to features at genetic level and allows unbiased phenotypic screens of many traits. With the cataloguing of measurable traits in this species, genetic rescue experiments can be successfully implemented. This could foster a new kind of conservation planning by promoting beneficial hybridization [14,15]. At present there are no entries for *C. retropictus* in the National Centre for Biotechnology Information (NCBI) records, which limits our understanding of functional transcripts from this species for application in prioritized conservation plans.

With the advent of next-generation sequencing (NGS) platforms, the genome and transcriptome of non-model species have been rapidly explored. A reduction in the cost of sequencing technologies has helped in the generation of a large quantity of genomic resources from endemic, endangered, and invasive species [16,17,18,19,20]. Transcriptomic information has helped in the study of the functional transcripts involved in biological, cellular, and molecular processes. Additionally, a number of valuable polymorphic microsatellite markers have been identified in relevant unigenes. The most recent transcriptomic characterization of molluscan species has used the Illumina HiSeq paired-end platform due to its relatively low cost and good results [19,21,22].

In this study, we present the first visceral mass transcriptomic analysis of the neritid *C. retropictus*, using the Illumina HiSeq 2500 platform, de novo analysis and annotation using protein and nucleotide sequences in public databases. The new datasets, which will be held publicly in a sequence database, should improve our understanding of genes involved in defense, immunity, and other metabolic processes. Furthermore, the discovery of microsatellite markers in the protein-coding gene sequences of this species will be valuable for analysing the attributes of functional genes in association with their phenotypes. The ultimate goal of this study is to describe the species transcriptome and provide genetic information for future studies that aim at understanding phenotypes, adaptation, inbreeding, and conservation priorities.

## 2. Materials and Methods

### 2.1. Ethics Statement

*C. retropictus* is regionally protected by law as endangered wildlife. Hence, permission for the use of samples of the species in the experiments was obtained from Yeongsangang River Basin Environmental Office (permission certificate number 2014-11; dated 10 July 2014). All animal experiments were conducted in accordance with national and international regulations.

### 2.2. Sample Collection and RNA Preparation

Four *C. retropictus* individuals were collected (collection date: 19 July 2014) from Sumun-ri, Anyang-myeon, Jangheung-gun, Jeollanam-do, South Korea. After transfer to the laboratory, the visceral mass tissue was carefully dissected and immediately placed in liquid nitrogen for RNA preparation. The flash-frozen visceral mass tissue was processed for total RNA using Trizol reagent (Invitrogen, Waltham, MA, USA) in accordance with the manufacturer’s instructions. Briefly, 1 mL of Trizol reagent was added per 50–100 mg of tissue samples. After the phase separation by adding chloroform and a subsequent centrifugation step, RNA was precipitated by adding 100% isopropanol. The RNA pellet was washed with 75% ethanol, vortexed, and vacuum-dried. The purity and concentration of RNA were measured using a NanoDrop-2000 spectrophotometer (Thermo Fisher Scientific, Waltham, MA, USA) and a Bioanalyzer 2100 system (Agilent Technologies, Santa Clara, CA, USA). Approximately 5 µg of total RNA pooled from the four sampled individuals was used as the input material for cDNA library construction.

### 2.3. Library Construction and Illumina Sequencing

cDNA libraries were constructed using Illumina’s “TruSeq RNA Sample Preparation Kit” (Illumina, San Diego, CA, USA) following the manufacturer’s recommendations. Briefly, mRNA was purified from total RNA using oligo (dT) magnetic beads and fragmented using divalent cations under an elevated temperature. First-strand cDNA was synthesized using random oligonucleotides and SuperScript II. Subsequently, second-strand cDNA synthesis was performed using DNA polymerase I, RNase H, buffer, and dNTPs. The double-stranded cDNA was end-repaired using T4 polynucleotide kinase (New England BioLabs, Ipswich, MA, USA), Klenow fragment (New England BioLabs), and T4 DNA polymerase (New England BioLabs). After end-repair of cDNA, Illumina paired-end (PE) adapter oligonucleotides were attached. The library fragments were purified with the AMPure XP system (Beckman Coulter, Beverly, MA, USA) and enriched by polymerase chain reaction (PCR). Finally, the library preparations were sequenced on an Illumina HiSeq 2500 platform at the sequencing facility of GnC Company (Daejeon, South Korea) with the generation of 100-base pair (bp) PE-reads. The datasets obtained were submitted to the NCBI Short Read Archive (SRA) database with the accession number SRP072407.

### 2.4. Quality Control of Reads and de novo Analysis

Raw reads from the sequencer in fastq format were pre-processed to obtain clean reads. Briefly, the adapter-only (recognized adapter lengths ≤ 13 nucleotides and remaining adapter excluded lengths ≤ 35 nucleotides) reads, reads containing poly-N, and low-quality reads were trimmed using the Sickle software (version 1.33) tool (http://github.com/najoshi/sickle) and Fastq_filter software (version 1.33) [23]. For de novo analysis, clean reads were assembled using the de Bruijin graph-based Trinity program [24], followed by the TIGR Gene Indices Clustering Tool 2.1 (TGICL) [25] with default parameters. This resulted in sequences called unigenes that could not be extended at either end. These are sequences that are expressed but not sufficiently characterized to be considered a gene.

### 2.5. Transcriptomic Annotation

We utilized multiple databases for the annotation of unigenes that could help to provide novel insights into functional transcripts. The databases used for functional annotation included public protein databases, such as the locally curated Protostome DB (PANM DB), Clusters of Orthologous Groups (COG), Gene Ontology (GO), and the Kyoto Encyclopaedia of Genes and Genomes (KEGG) database, as well as the Unigene nucleotide database (a cluster of sequences within the NCBI database). The query unigene sequences were then matched with the subject sequences in the multiple databases using BLAST (BLASTX tool for proteins and BLASTN tool for nucleotides) at an E-value cut-off of e-5 (<0.00001) [26]. Subsequently, GO analysis was performed based on PANM DB annotation using the BLAST2GO professional version software (http://www.BLAST2go.org/). WEGO software (http://wego.genomics.org.cn/cgi-bin/wego/index.pl/) was utilized for the classification of unigenes into GO term (level 2) categories [27]. The classification of unigenes (including those with Enzyme Commission Numbers) into the functional pathways was conducted by KEGG analysis [28]. The KEGG automatic annotation server was used for KEGG Orthology (KO) and KEGG pathway annotation. Similarly, a BLASTX search against the COG database resulted in the classification of unigenes into COG functional groups [29].

### 2.6. Identification of Simple Sequence Repeats (SSRs)

Microsatellites in unigenes were screened using the MicroSAtellite Identification Tool (MISA; version 2.0) available at http://pgrc.ipk-gatersleben.de/misa/. For the identification of SSRs, only unigenes with a length of >1000 bp were selected. Minimum unit size cut-offs of six, five, four, four, and four were used to report di-, tri-, tetra-, penta-, and hexanucleotide repeats, respectively. Mononucleotide repeats were excluded from the analysis due to the possibility of homopolymer generation during Illumina sequencing. A minimum distance of 100 nucleotides was allowed between two SSRs. Furthermore, for SSR validation in the detection of polymorphism, primers were designed with the following criteria: dinucleotides with a minimum of six iterations and tri-/tetranucleotides with a minimum of four iterations. The primers designed using the BatchPrimer 3 program [30] show the following features: primer size of 18–23 bases with an optimum size of 21 bases, product size of 100–300 bases, melting temperature (Tm) ranging from 50–70 °C, and GC% of 30%–70%.

## 3. Results and Discussion

### 3.1. Illumina Reads and Sequence Assembly

**Figure 1:**
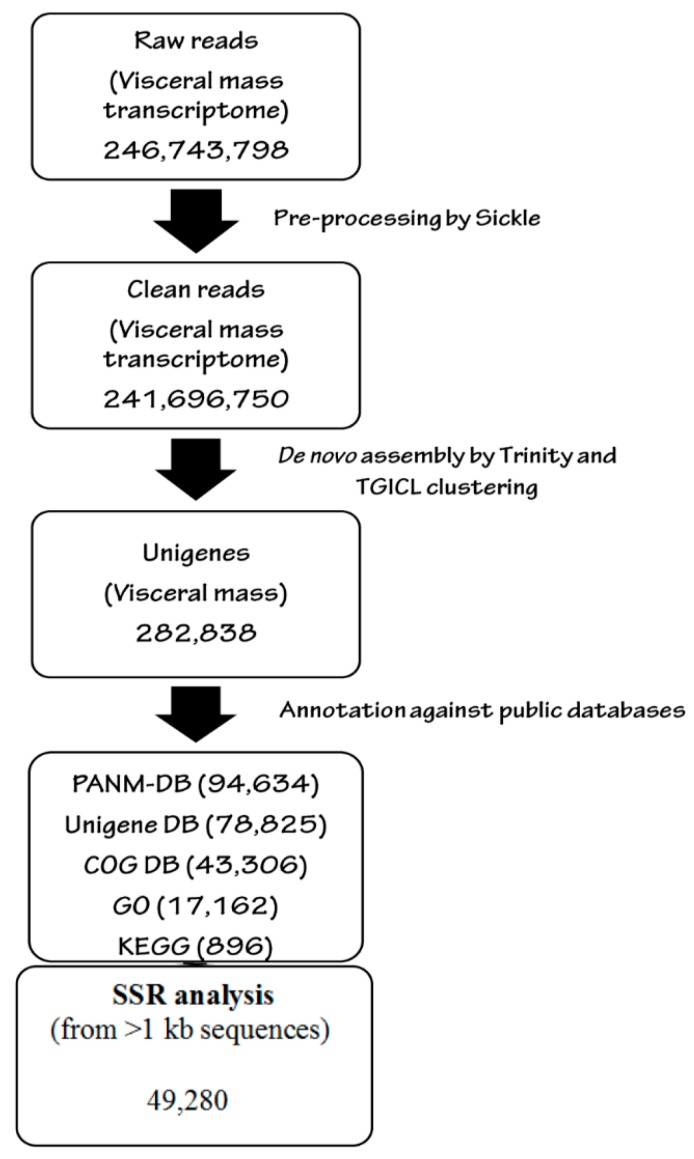
Schematic work-flow of *C. retropictus* transcriptome using Illumina sequencing, de novo analysis, and annotation. The visceral mass transcriptome of *C. retropictus* was obtained using an Illumina HiSeq 2500 platform. The raw reads were pre-processed using the Sickle software tool (quality: 20, length: 40) and Fastq_filter software to obtain clean reads. Using Trinity (K-mer: 25; minimum contig length: 200) de novo assembler and TGICL clustering, the clean reads were processed to unigene sequences. Subsequently, the unigene sequences were blasted against public databases including PANM, Unigene, COG, GO, and KEGG for functional annotation. The SSRs were detected within the unigene sequences using MISA software.

We obtained the first visceral mass transcriptome of *C. retropictus*, an endangered mollusc of the neritid family found only in restricted locations in South Korea. The *C. retropictus* transcriptome was de novo assembled using Trinity software with the default parameters (sequence length > 200 nucleotides). The overall scheme of the whole-transcriptome study of *C. retropictus* is shown in Figure 1. The overall number of Illumina read pairs for the sample was 123,371,899 (246,743,798 sequences with 31,089,718,548 nucleotides processed), and they were archived with NCBI with the accession number SRP072407. Furthermore, the sequencing information (including the assembled contigs) can be downloaded from http://bioinfo.sch.ac.kr/submission/ one year from the date of submission (26 March 2016). The raw reads were subjected to quality control, which included trimming of adapter sequences using the Cutadapt program. We recovered 99.80% of reads with an average length of 125.8 bp after the adapter trimming process. After removing the adapter and low-quality sequences, we obtained 30,038,741,871 clean read bases (241,696,750 sequences). The processed sequences were used for de novo assembly. An overview of pre-processing of the raw reads is shown Table S1.

**Figure 2:**
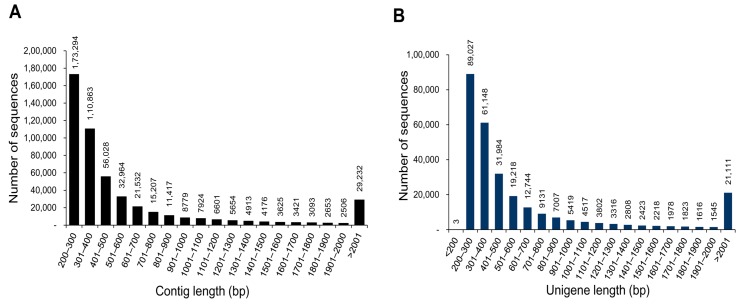
Sequence length distribution of de novo assembled contigs (**A**) and unigenes (**B**) from *C. retropictus* transcriptome.

A total of 503,882 contigs having 330,800,003 bases were identified. The contigs had an average and N50 length of 656.5 and 953 bp, respectively with the largest contig of 22,900 bp. The size distribution of contigs showed that 340,185 contigs had lengths < 500 nucleotides, 89,899 had lengths in between 501 and 1000 bp, 29,268 had lengths between 1001 and 1500 bp, 15,298 had lengths in between 1501 and 2000 bp, and 29,232 had lengths > 2001 bp (Figure 2A). The average and N50 lengths of the assembled contigs obtained in the present study were longer than those previously reported for the visceral mass transcriptome of the bradybaenid gastropods *Aegista chejuensis* (average and N50 length of 610.8 bp and 788 bp, respectively) and *Aegista quelpartensis* (average and N50 length of 582.1 bp and 719 bp, respectively) [19]. The average and N50 lengths were also longer than those of the multiple-tissue transcriptome of *Anadara trapezia* [31], the whole transcriptome of *Echinolittorina malaccana* [32], and the mantle transcriptome of *Chlamys farreri* [33]. Furthermore, we used TGICL for clustering the contigs to form a single pair of non-redundant unigenes. Clustering reduces the redundancy of the sequences and enriches the information contained in the cluster. We obtained a total of 282,838 unigenes having 208,418,920 bases with an average and N50 length of 736.9 and 1201 bp, respectively. The N50 value is one of the assessed parameter for understanding the quality of the assembly. The high N50 value for unigenes obtained in this study is better than that for transcriptome assemblies in invertebrates [34,35,36]. By TGICL clustering, the smallest unigene showed a length of 110 bp, whereas the length of the largest one was 22,900 bp. The size distribution of unigenes is shown in Figure 2B. Approximately 64.4% of the unigenes had lengths < 500 bp, 18.92% had lengths ranging between 501 and 1000 bp, 7.46% had lengths > 2000 bp, 5.90% had lengths in between 1000 and 1500 bp, and 3.25% had lengths between 1501 and 2000 bp. A negative trend (longer unigenes = less sequences) was observed between the unigene length and the number of sequences. The summary statistics of *C. retropictus* transcriptome sequencing and assembly are shown in Table 1.

### 3.2. Annotation of Unigenes and BLAST-Based Homology Search

**Figure 3:**
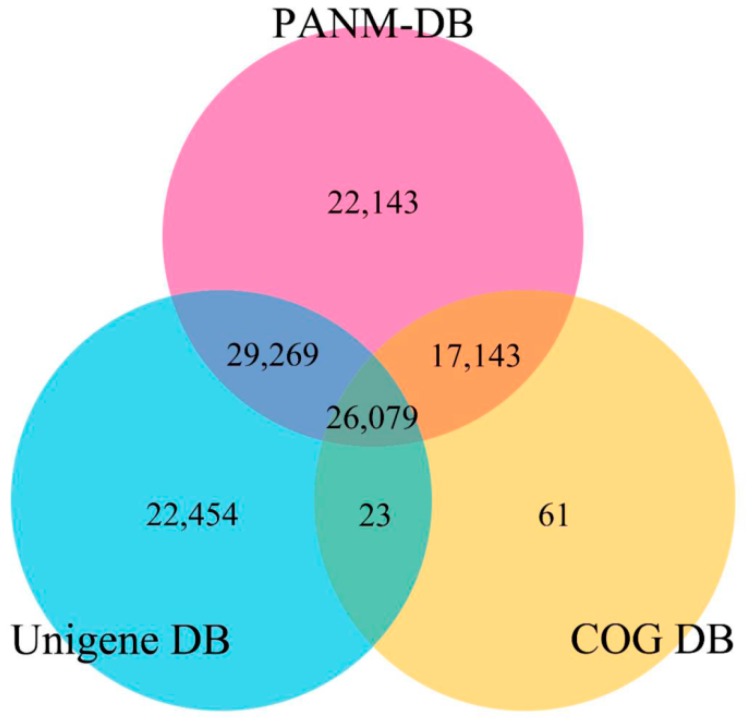
The annotation of *C. retropictus* unigenes against the protein databases PANM and COG and the nucleotide database Unigene using BLASTX and BLASTN (E-value ≤ 1 × 10^−5^), respectively.

We attempted to annotate the sequences of *C. retropictus* unigenes using five different databases. PANM-DB was preferred over the NCBI nr database due to its speed and efficiency of annotation retrieval. The processing of annotation data by PANM-DB was 15 times faster, with twice as many significant hits compared with the NCBI nr database [37]. PANM-DB is considered valuable for annotating NGS data on non-model species belonging to a majority of the Protostomia group as it resolves the inconsistencies in molluscan sequences available at NCBI [19,20]. We used the BLASTX program at a stringency of E ≤ 0.000001 to annotate unigene sequences against the protein databases, such as PANM, COG, GO, and KEGG, and used the BLASTN program with a similar E-value cut-off to annotate sequences against Unigene DB. Of 282,838 unigenes, 125,616 sequences (44.41% of the total) showed a match to at least one of the databases used for annotation; 94,634 (33.46% of the total), 78,825 (27.87%), and 43,306 unigenes (15.31%) matched in the PANM, Unigene, and COG databases, respectively. In total, 17,162 (6.07% of the total) and 896 (0.32%) unigenes showed homology to matches in the GO and KEGG functional databases, respectively. The annotation of *C. retropictus* unigene sequences against protein and nucleotides sequences in public databases is shown in Table 2. Although a greater proportion of the unigene sequences annotated against PANM and Unigene DB showed lengths ranging from 300 to 1000 bp, most unigenes annotated against COG DB had lengths ≥ 1000 bp. Approximately 55.59% of unigenes did not show homology to known proteins in the five databases, as noted in molluscan transcriptomic studies [19,38]. These sequences probably represent untranslated mRNA regions, transcripts lacking conserved protein domains or transcripts derived from assembly errors. We also speculate that some of these sequences would represent novel genes not reported before and hence unique to this species [36,38,39]. Furthermore, to understand the annotation of unigenes to homologous sequences in PANM, Unigene, and COG DB, we constructed a three-way Venn diagram (Figure 3). Among the total unigenes annotated in PANM DB, 30.93% and 18.12% showed annotation with Unigene and COG DB, respectively. Approximately, 27.56% of unigenes had homologous matches in three of the databases, whereas 23.40% of unigenes were uniquely annotated to only PANM DB. Similarly, 28.49% of the assembled sequences found matches to homologous sequences in Unigene DB, and only 0.14% found matches in COG DB. Additionally, there was a 39.6% overlap of sequence annotations in COG DB with those in PANM DB with regard to the *C. retropictus* transcriptome. The details of unigene annotation against PANM, Unigene, and COG DB is shown in Table S2.

**Figure 4:**
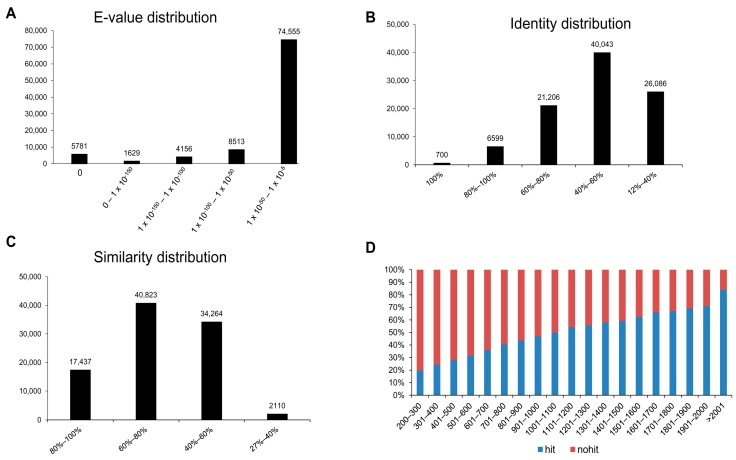
Homology search statistics of assembled unigenes against PANM protein database. (**A**) E-value distribution of each unigene using BLAST with a cut-off E-value of 1 × 10^−5^; (**B**) identity distribution of the top BLAST hits for each unigene; (**C**) similarity distribution of top BLAST hits for each unigene; (**D**) the ratio of BLAST hits vs. non-hits and its relationship with the length of transcripts.

The unigene sequences annotated against PANM DB using the BLASTX program were assessed for the top-hit E-values, identity distribution, similarity distribution, and hit and non-hit ratio (Figure 4). The E-value distribution of the annotated unigenes showed that the top-hit E-value ranged from 1 × 10^−50^ to 1 × 10^−5^ (79% of sequences). Other top-hit E-value distribution ranges were less than 10% for unigene sequences (Figure 4A). We found that 42% of unigene sequences had identities of 40%–60% to known proteins in the database, followed by 28% and 22% of unigenes having identities of 12%–40% and 60%–80%, respectively (Figure 4B). Furthermore, 43% of sequences showed similarity of 60%–80% and 36% showed similarity of 40%–60% to known sequences in the database (Figure 4C). There was also a direct relationship between the annotation hits and the length of the sequences. Sequences having a length of less than 500 bp were less frequently annotated (less than 30%) compared with those with more frequent annotations (Figure 4D). BLAST-based sequence annotation involves conserved domain-specific analysis and, therefore, shorter sequences may define no or uncharacteristic domains for homology.

**Figure 5:**
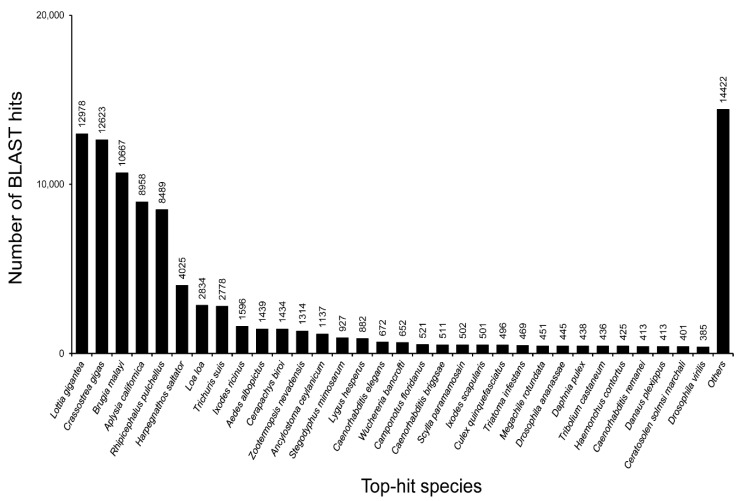
Species distribution analysis for *C. retropictus* transcriptome based on top-BLAST hits against PANM protein database.

With regard to the top-hit species distribution, for matching against PANM DB, 13.71% of the assembled sequences showed similarities with *Lottia gigantea*; this was followed by *Crassostrea gigas* (13.34% of the assembled sequences) (Figure 5). *L. gigantea* (owl limpet) is a gastropod snail and the first lophotrochozoan for which a draft genome has been characterized; it has helped in the prospecting of genes for many functional processes [40]. Similarly, analyses of the genome along with the transcriptome of *C. gigas* (Pacific oyster) have revealed an extensive set of genes for environmental stress and adaptation, thus ensuring a thorough understanding of lophotrochozoans. Approximately 9.47% of the assembled sequences showed top-hit homology to *Aplysia californica* genes, for which the genomic resources have been characterized in detail [41].

### 3.3. COG, GO, and KEGG Classifications

**Figure 6:**
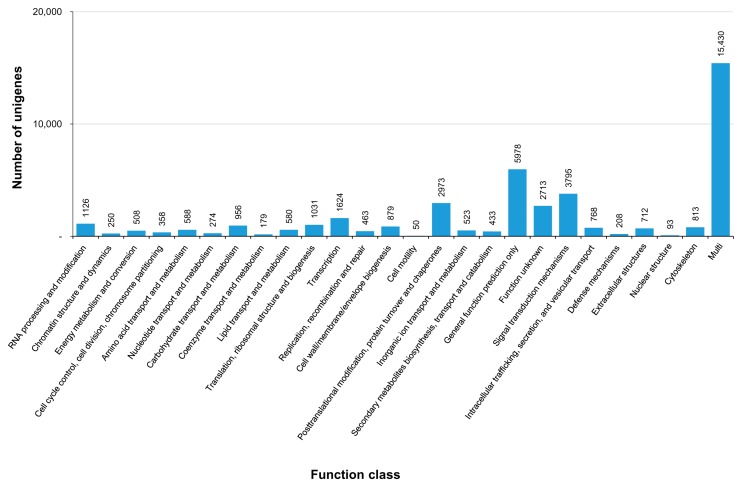
COG classification of *C. retropictus* unigenes.

For the functional prediction, the assembled unigenes were subjected to a BLASTX search against the proteins in the COG database. COG represents an orthologous gene product database wherein every protein is assumed to have evolved from an ancestral protein, and the whole database is built on coding proteins. The classification of the unigenes into 26 COG classes is shown in Figure 6. Of the COG annotated unigenes, most (35.63%) were clustered under the ‘multi’ functional group, followed by 13.8%, 8.76%, 6.87%, and 5.02% under general function prediction; signal transduction mechanisms; post-translational modification, protein turn-over, and chaperones; and unknown function, respectively. Among the COG functional categories, nuclear structure and cell motility were the least represented. The COG-based functional prediction of *C. retropictus* unigenes were found to be consistent with the COG representation of unigenes in the bradybaenid snails of the genus *Aegista* [19].

**Figure 7:**
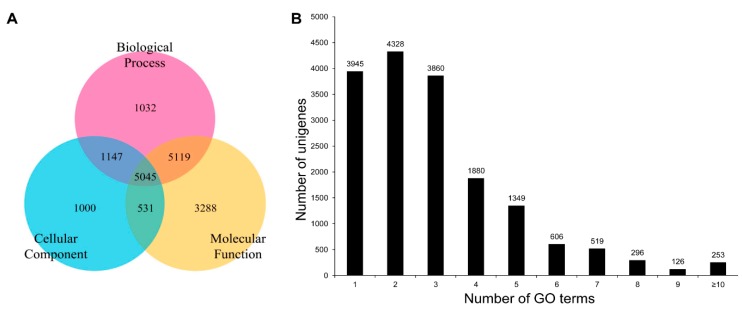
Gene Ontology (GO) database-based annotations. (**A**) A three-way Venn diagram suggesting the annotation of unigenes to three major GO categories as biological process, cellular component, and molecular function; (**B**) the representation of unigenes under one or more GO terms.

**Figure 8:**
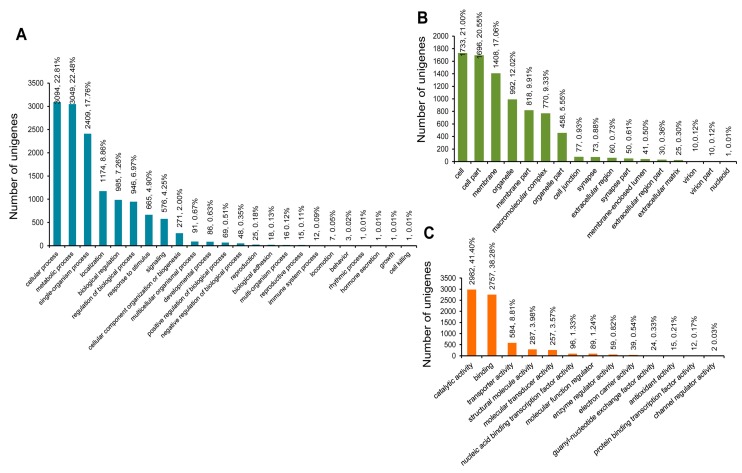
The top represented GO term categories in each of the three major GO domains. (**A**) Biological process; (**B**) cellular component; and (**C**) molecular function.

The assembled sequences of *C. retropictus* were classified into GO categories and KEGG pathways using the BLAST2GO (B2G) bioinformatics tool [42]. B2G was originally developed as a web-interface for GO consortium annotation, but it subsequently included functions such as Enzyme Code (EC), KEGG Maps, and InterPro Motifs [43]. Generally, under the B2G tool, BLASTX is run at NCBI followed by an InterProScan analysis and subsequent GO mapping. The assignment of each query sequence into three GO functional categories, namely biological process, cellular component, and molecular function, is associated with evidence codes (ECs) that define the validity of the functional annotation [44]. In the present study, 17,162 unigene sequences were annotated using the GO database and assigned to the three functional categories. A three-way Venn chart summarizes the assignment of the GO-annotated unigenes to biological process (1032 unigenes), cellular component (1000 unigenes), and molecular function categories (3288 unigenes). Furthermore, 5119 sequences were assigned to both biological process and molecular function and 5045 sequences were assigned to all three GO functional categories (Figure 7A). The detailed information on the suggested classification of unigenes to biological process, molecular function, and cellular component category is shown in Table S3. We find that only 22.99% of unigene sequences were annotated to a single GO term, and the rest were ascribed to more than one GO term annotation. A histogram depicting the distribution of the unigenes regarding the number of GO terms is shown in Figure 7B. The results of assignment of the unigenes to three GO categories (level 2) are shown in Figure 8. For the biological process category, the predominant GO terms included “cellular process” (3094; 22.81%), “metabolic process” (3049, 22.48%), and “single-organism process” (2409, 17.76%) (Figure 8A). In the cellular component process, “cell” (1733; 21%), “cell part” (1696; 20.55%), “membrane” (1408; 17.06%), and “organelle” (992, 12.02%) represented the predominant GO terms for the classification of unigenes (Figure 8B). Meanwhile, the unigenes were assigned to 13 terms in the molecular function process, the predominant one being “catalytic activity” (2982; 41.40%); this was closely followed by “binding” (2757; 38.28%) (Figure 8C). The GO term annotation results only suggest a gene grouped to a known (or predictive) function, and are, thus, not evidence of functionality [45,46,47]. The EC associated with each GO term annotation suggests a majority of terms to be of “IEA” (inferred from electronic annotation) quality, which is not manually curated or experimentally verified. In this scenario, the interpretation of unigene function is only predictive.

**Figure 9:**
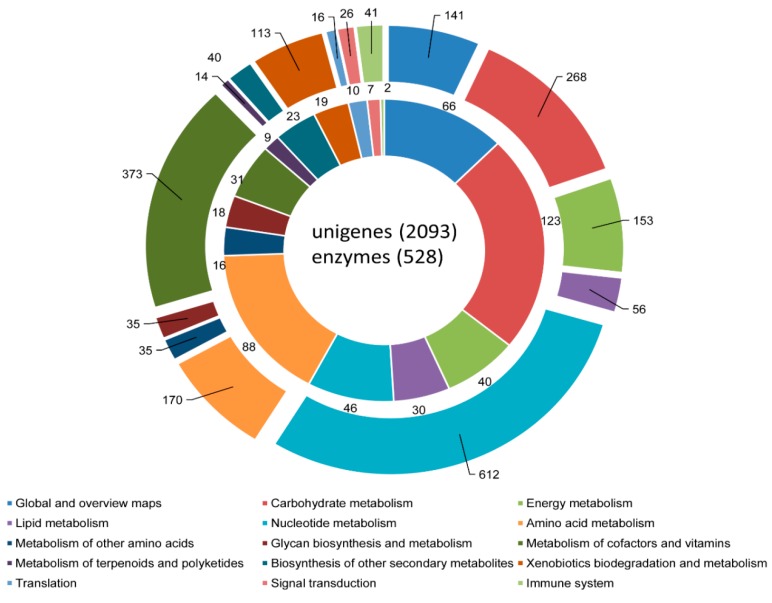
KEGG pathway analysis. The *C. retropictus* visceral mass unigenes were assigned to KEGG pathways (outer circle). The sequences with the Enzyme Commission (EC) numbers ascribed under each KEGG pathway are shown in the inner circle. Each pathway is represented by a different color.

KEGG pathway analysis helps to record the biological functions of genes through elucidation of enzymes and other proteins in biochemical processes [28,48]. It is essentially an enrichment process for the classification of genes to functional processes such as “metabolism”, “environmental information processing”, “genetic information processing” and “cellular processes”. In the present study, 2093 unigene sequences (including 528 sequences with EC numbers) were classified to 107 pathways KEGG pathways (Table S4). As expected, most of the sequences were assigned to the “metabolism” category, with “carbohydrate metabolism” forming the dominant group, with 268 unigenes (including 123 sequences with EC numbers), followed by nucleotide metabolism, with 612 sequences (including 46 sequences with EC numbers). Other important KEGG pathways to which the unigene sequences were assigned included “translation”, “signal transduction” and “immune system”, with 16, 26, and 41 sequences, respectively. The representative pathways from KEGG pathway annotation are depicted in Figure 9. Overall, the COG, GO, and KEGG pathways were sufficient to predict the functionality of annotated sequences at the whole-transcriptome level. This putative functional analysis should be useful in the path toward experimental validation of gene function through a targeted gene approach. The immune-, defense-, and reproduction-related transcripts identified from such transcriptomics studies, in particular, have been utilized to shed light on the resistance of organisms to biotic and abiotic stresses, adaptation potential, and commercial success [49,50,51].

### 3.4. InterProScan Analysis for Conserved Protein Domains

An InterProScan using the B2G bioinformatics tool searched for conserved protein domains in the unigene sequences obtained from *C. retropictus* transcriptomic analysis. Altogether, 21,069 sequences (7.45% of the total unigenes) showed conserved InterPro domains. A list (top 20) of the most abundant InterPro domains is shown in Table 3. This includes the zinc fingers (C2H2-like and RING-type), ankyrin repeats, immunoglobulin subtype, protein kinases (serine/threonine/dual specificity), EGF-like, RNA recognition motif, WD40, and C-type lectin domain. Protein kinases form one of the most abundant domains, and the related proteins are involved in intracellular cell signaling, metabolism, transcription, differentiation, and apoptosis. The kinases are pivotal in modulating the NF-ĸB signaling in the invertebrate immune system [52]. Zinc finger domains are abundant in protein sequences and are responsible for interactions with DNA, RNA, and proteins [53]. The zinc finger domains are very highly conserved and have been identified in many molluscan transcriptomes [20,38,54,55]. C-type lectins are pathogen recognition molecules having one or more carbohydrate recognition domains (CRDs). These act as one of the non-self-determinants conferring immunomodulatory signals in molluscs [56]. InterProScan analysis thus adds to the functional annotation of unigenes, and conserved protein domain searches could reveal the putative functions of assembled sequences.

### 3.5. SSR Characterization and Primer Identification

Next-generation sequencing methods have shown immense potential in unraveling polymorphic microsatellites for studies focusing on species diversity, population dynamics, and conservation genetics. As microsatellites are primarily identified in unigene sequences putatively related to a protein function, these markers could be utilized for large-scale gene polymorphism studies, facilitating evolutionary analyses [48]. In the present study, we screened SSRs in 48,973 unigene sequences (106,535,022 bases) having lengths of ≥1 kilobase. Using the MISA tool, a total of 49,280 potential SSRs were screened. These SSRs were identified from 25,365 sequences (51.79% of total unigene sequences considered), with 12,546 sequences containing more than one SSR. Of the identified SSRs, 68.42% were di-nucleotide repeats; this was followed by 16.99% tri-nucleotide repeats and 14.59% tetra/penta/hexa-nucleotide repeats (Table 4). The predominance of di-nucleotide repeats in the unigene sequences of *C. retropictus* is in agreement with findings of SSRs in invertebrates, including molluscs [12,50,57]. Mononucleotide repeats were not included in the SSR screening as they may be the result of homopolymer generation, which is common in Illumina sequencing. Di-nucleotide, tri-nucleotide, and tetra-nucleotide repeats were represented most commonly in six (22.88% of repeats), five (49.74%), and four (60.13%) iterations, respectively (Table 5).

**Figure 10:**
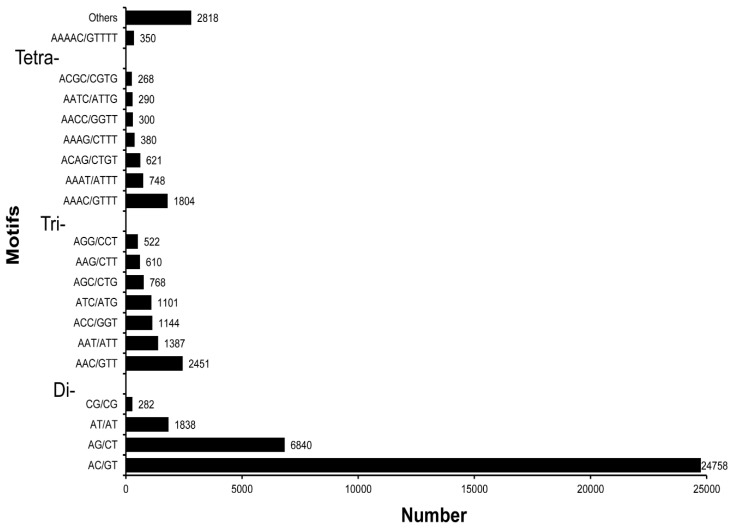
The distribution of SSR repeats motifs found in *C. retropictus* visceral mass unigenes. For an analysis of types of repeats and motifs, only unigenes having >1 kb sequence size were considered.

Among the dinucleotide repeats, (AC/GT)n, (AG/CT)n, and (AT/AT)n were the three predominant repeat motif types, with frequencies of 73.43%, 20.29%, and 5.45%, respectively. Among the tri-nucleotide repeats, (AAC/GTT)n, followed by (AAT/ATT)n, and (ACC/GGT)n, were the most common types, with a combined frequency of 59.5%. Similarly, among the tetra-nucleotide repeats, (AAAC/GTTT)n was the most predominant, with a frequency of 31.34%. The distribution of repeat motif types is shown in Figure 10. We also attempted to identify PCR primers flanking the SSR regions for further validation of polymorphic sites using Primer 3. Table S5 shows the characteristics of primers that can be used for the validation of SSRs identified from the transcriptomic study. To date, microsatellites have not been explored from *C. retropictus,* and, hence, the development of SSRs for the protection of this species in its natural habitat is highly desirable. Natural populations of the endangered species can be augmented by beneficial hybridization or supportive breeding. In this regard, microsatellites are important genetic tools to assess the loss of genetic diversity that contributes to extinction risks, especially in smaller populations of endangered species [58].

## 4. Conclusions

Here, we report the first comprehensive transcript dataset of *Clithon retropictus*, a brackish-water snail classified as an endangered class II species in South Korea. In total, 282,838 unigenes were annotated for putative functions using protein and nucleotide databases. This will enable the identification of immunity-, defense-, and reproduction-related genes, which should facilitate approaches to the conservation of this species in the wild. Furthermore, the large number of identified SSRs provides targets for identifying polymorphisms across the populations. In terms of the design of informed conservation plans for the species, the transcriptome provides an amount of data that circumvents the need to decide on a limited number of traits and allows unbiased phenotypic screens of many traits.

## Figures and Tables

**Table 1:** Statistical summary of *C. retropictus* transcriptome and de novo assembly.

**Transcriptome Summary**	
Number of raw read sequences	246,743,798
Number of raw read bases	31,089,718,548
Number of clean read sequences	241,696,750
Number of clean read bases	30,038,741,871
Mean length of contig (bp)	124.3
N50 length of contig (bp)	126
GC % of contig	50.93
High-quality reads (%)	97.95 (sequences), 96.62 (bases)
**Contig Information**	
Total number of contigs	503,882
Number of bases	330,800,003
Mean length of contig (bp)	656.5
N50 length of contig (bp)	953
GC % of contig	46.80
Largest contig (bp)	22,900
No. of large contigs (≥500 bp)	164,129
**Unigene Information**	
Total number of unigenes	282,838
Number of bases	208,418,920
Mean length of unigene (bp)	736.9
N50 length of unigene (bp)	1201
GC % of unigene	46.61
Length ranges (bp)	110–22,900

**Table 2:** Sequence annotation of *C. retropictus* unigenes against public databases.

Database	All	≤300 bp	300–1000 bp	≥1000 bp
PANM	94,634	17,610	43,881	33,143
Unigene	78,825	16,601	38,056	24,168
COG	43,306	5916	15,559	21,831
GO	17,162	3702	7915	5545
KEGG	896	138	240	518
All Database	125,616	26,229	61,447	37,940

**Table 3:** The top 20 InterPro domains in the unigenes of *C. retropictus*.

Domain	Short Name	Description	Number of Unigenes
IPR015880	Znf_C2H2-like	Zinc finger, C2H2-like domain	1141
IPR002110	Ankyrin_rpt	Ankyrin repeat	862
IPR027417	P_loop_NTPase	P-loop containing nucleoside triphosphate hydrolase domain	566
IPR013783	Ig-like_fold	Immunoglobulin-like fold domain	475
IPR020683	Ankyrin_rpt-contain_dom	Ankyrin repeat-containing domain	360
IPR000477	RT_dom	Reverse transcriptase domain	338
IPR003599	Ig_sub	Immunoglobulin subtype domain	307
IPR000504	RRM_dom	RNA recognition motif domain	271
IPR002290	Ser/Thr_dual-sp_kinase	Serine/threonine/dual specificity protein kinase, catalytic domain	257
IPR000742	EGF-like_dom	EGF-like domain	244
IPR011989	ARM-like	Armadillo-like helical domain	206
IPR005135	Endonuclease/exonuclease/ phosphatase	Endonuclease/exonuclease/phosphatase domain	202
IPR000276	GPCR_rhodopsn	G protein-coupled receptor, rhodopsin-like family	198
IPR001680	WD40_repeat	WD40 repeat	187
IPR003598	Ig_sub2	Immunoglobulin subtype 2 domain	181
IPR001841	Znf_RING	Zinc finger, RING-type domain	176
IPR002048	EF_hand_dom	EF-hand domain	161
IPR001881	EGF-like_Ca-bd_dom	EGF-like calcium-binding domain	160
IPR001304	C-type_lectin	C-type lectin domain	159
IPR002035	VWF_A	von Willebrand factor, type A domain	158

**Table 4:** Identification of SSRs and repeat types from unigene sequences (>1 kb) of *C. retropictus* transcriptome.

*SSR parameters*	*Numbers identified*
*Total number of sequences examined*	48,973
*Total size of examined sequences (bp)*	106,535,022
*Total number of identified SSRs*	49,280
*Number of SSR containing sequences*	25,365
*Number of sequences containing more than 1 SSR*	12,546
*Number of SSRs present in compound formation*	11,278
*Unit size*	Number of SSRs
*2*	33,718
*3*	8373
*4*	5756
*5*	1180
*6*	253

**Table 5:** Summary of SSRs and their repeat numbers in *C. retropictus* unigenes of length > 1 kb.

Repeats	4	5	6	7	8	9	10	11	12	13	14	15	16	17	18	19	20	≥21	Total
Di	0	0	7713	5048	3566	2579	2119	2543	2180	676	811	710	614	556	504	479	435	3185	33,718
Tri	0	4165	1899	940	729	123	139	74	51	43	26	28	35	28	20	10	23	40	8373
Tetra	3461	1263	680	91	77	51	29	15	28	15	6	6	8	6	3	4	0	13	5756
Penta	740	220	39	34	25	27	27	17	13	11	7	11	3	2	0	0	0	4	1180
Hexa	222	18	9	1	1	2	0	0	0	0	0	0	0	0	0	0	0	0	253
Total	4423	5666	10,340	6114	4398	2782	2314	2649	2272	745	850	755	660	592	527	493	458	3242	49,280

## Supplementary Materials

The following are available online at www.mdpi.com/2073-4425/7/7/35/s1, Table S1: Pre-processing of raw reads using the Cutadapt program; Table S2: The annotation of unigenes against PANM, Unigene and COG databases; Table S3: The suggested classification of unigenes to GO functional groups. C, cellular component; F, molecular function; P, biological process; Table S4: KEGG biochemical pathway mapping for *C. retropictus* unigenes; Table S5: The primers flanking the SSR polymorphic sites of *C. retropictus* unigenes.

## References

[1] Neubauer T., Schneider S., Bohme M., Prieto J. (2012). First records of freshwater rissooidean gastropods from the paleogene of South-East Asia. J. Mollusc. Stud..

[2] Chukwuka C.O., Ejere V.C., Asogwa C.N., Nnamonu E.I., Okeke O.C., Odii E.I., Ugwu G.C., Okanya L.C., Levi C.A. (2014). Eco-physiological adaptation of the land snail *Achatina achatina* (Gastropoda: Pulmonata) in tropical agro-ecosystem. J. Basic Appl. Zool..

[3] Hayes K.A., Cowie R.H., Jorgensen A., Schultheib R., Albrecht C., Thiengo S.C. (2009). Molluscan studies in evolutionary biology: Apple snails (Gastropoda: Ampullariidae) as a system for addressing fundamental questions. Am. Malac. Bull..

[4] Amin S., Prentis P.J., Gilding E.K., Pavasovic A. (2014). Assembly and annotation of a non-model gastropod (*Nerita melanotragus*) transcriptome: A comparison of de novo assemblers. BMC Res. Not..

[5] Ohara T., Tomiyama K. (2000). Niche segregation of coexisting two freshwater snail species *Semisulcospira libertine* (Gould, Prosobranchia: Pleuroceridae) and *Clithon retropictus* Martens, Prosobranchia: Neritidae). Jpn. J. Malacol..

[6] Furojo Y., Tomiyama K. (2000). Distribution and microhabitat of coexisting two freshwater snail species, *Semisulcospira libertine* (Gould, Prosobranchia: Pleuroceridae) and *Clithon retropictus* (Martens, Prosobranchia: Neritidae). Jpn. J. Malacol. Venus.

[7] Noseworthy R.G., Lee H.-J., Choi K.-S. (2013). The occurrence of *Clithon retropictus* (v. Martens, 1879) (Gastropoda: Neritidae) in an unusual habitat, Northern Jeju Island, Republic of Korea. Ocean Sci. J..

[8] Miyajima H., Wada K. (2014). Spatial distribution in relation to life history in the neritid gastropod *Clithon retropictus* in the Kanzaki River Estuary, Osaka, Japan. Plankton Benthos Res..

[9] Kwon O.K., Park G.M., Lee J.S. (1993). Colored Shells of Korea.

[10] Kwon O.G., Min D.K., Lee J.R., Lee J.S., Je J.G., Choe B.L. (2001). Korean Mollusks with Color Illustrations.

[11] Min D.K. (2004). Mollusks in Korea.

[12] Noseworthy R.G., Ju S.J., Choi K.S. (2012). The occurrence of *Clithon retropictus* (von Martens in Kobelt, 1879, Gastropoda: Neritidae) in Jeju Island, Republic of Korea. Korean J. Malacol..

[13] Malmqvist B., Rundle S. (2002). Threats to the running water ecosystems of the world. Environ. Conserv..

[14] Primm S.L., Dollar L., Bass O.L. (2006). The genetic rescue of Florida panther. Animal Conserv..

[15] Ellstrand N.C., Biggs D., Kaus A., Lubinsky P., McDade L.A., Preston K., Prince L.M., Regan H.M., Rorive V., Ryder O.A. (2010). Got hybridization? A multidisciplinary approach for informing science policy. BioScience.

[16] Wang X.W., Zhao Q.Y., Luan J.B., Yan G.H., Liu S.S. (2012). Analysis of a native whitefly transcriptome and its sequence divergence with two invasive whitefly species. BMC Genomics.

[17] Ioannidis P., Lu Y., Kumar N., Creasy T., Daugherty S., Chibucos M.C., Orvis J., Shetty A., Ott S., Flowers M. (2014). Rapid transcriptome analysis of an invasive pest, the brown marmorated stink bug *Halyomorpha halys*. BMC Genomics.

[18] Richardson M.F., Sherman C.D.H. (2015). De novo assembly and characterization of the invasive Northern Pacific Seastar transcriptome. PLoS ONE.

[19] Kang S.W., Patnaik B.B., Hwang H.-J., Park S.Y., Wang T.H., Park E.B., Chung J.M., Song D.K., Patnaik H.H., Lee J.B. (2016). De novo transcriptome generation and annotation for two Korean endemic land snails, *Aegista chejuensis* and *Aegista quelpartensis*, using Illumina paired-end sequencing technology. Int. J. Mol. Sci..

[20] Patnaik B.B., Wang T.H., Kang S.W., Hwang H.-J., Park S.Y., Park E.B., Chung J.M., Song D.K., Kim C., Kim S. (2016). Sequencing, de novo assembly, and annotation of the transcriptome of the endangered freshwater pearl bivalve, *Cristaria plicata*, provides novel insights into functional genes and marker discovery. PLoS ONE.

[21] Zhang J., Liang S., Duan J., Wang J., Chen S., Cheng Z., Zhang Q., Liang X., Li Y. (2012). De novo assembly and characterization of the transcriptome during seed development, and generation of genic-SSR markers in Peanut (*Arachis hypogea* L.). BMC Genomics.

[22] Castellanos-Martinez S., Arteta D., Catarino S., Gestal C. (2014). De novo transcriptome sequencing of the *Octopus vulgaris* hemocytes using Illumina RNA-Seq technology: Response to the infection by the gastrointestinal parasite *Aggregata octopiana*. PLoS ONE.

[23] Blankenberg D., Gordon A., von Kuster G., Coraor N., Taylor J., Nekrutenko A. (2010). The Galaxy Team. Manipulation of FASTQ data with Galaxy. Bioinformatics.

[24] Grabherr M.G., Haas B.J., Yassour M., Levin J.Z., Thompson D.A., Amit I., Adiconis X., Fan L., Raychowdhury R., Zeng Q. (2011). Full-length transcriptome assembly from RNA-Seq data without a reference genome. Nat. Biotechnol..

[25] Pertea G., Huang X., Liang F., Antonescu V., Sultana R., Karamycheva S., Lee Y., White J., Cheung F., Parvizi B. (2003). TIGR Gene Indices Clustering Tool (TGICL): A software system for fast clustering of large EST datasets. Bioinformatics.

[26] Camacho C., Coulorious G., Avagyan V., Ma N., Papadopoulos J., Bealer K., Madden T.L. (2009). BLASTX+ architectures and structure. BMC Bioinform..

[27] Ye J., Fang L., Zheng H., Zhang Y., Chen J., Zhang Z., Wang J., Li S., Li R., Bolund L. (2006). WEGO: A web tool for plotting GO annotations. Nucl. Acids Res..

[28] Kanehisa M., Goto S. (2000). KEGG: Kyoto encyclopaedia of Genes and Genomes. Nucl. Acids Res..

[29] Tatusov R.L., Fedorova N.D., Jackson J.D., Jacobs A.R., Kiryutin B., Koonin E.V., Krylov D.M., Mazumder R., Mekhedov S.L., Nikolskaya A.N. (2003). The COG database: An updated version including eukaryotes. BMC Bioinform..

[30] You F.M., Huo N., Gu Y.Q., Luo M.-C., Ma Y., Hane D., Lazo G.R., Dvorak J., Anderson O.D. (2008). BatchPrimer 3: A high throughput web application for PCR and sequencing primer design. BMC Bioinform..

[31] Prentis P.J., Pavasovic A. (2014). The *Anadara trapezia* transcriptome: A resource for molluscan physiological genomics. Mar. Genomics.

[32] Wang W., Hui J.H.L., Chan T.F., Chu K.H. (2014). De novo transcriptome sequencing of the snail *Echinolittorina malaccana*: Identification of genes responsive to thermal stress and development of genetic markers for population studies. Mar. Biotechnol..

[33] Shi M., Lin Y., Xu G., Xie L., Hu X., Bao Z., Zhang R. (2013). Characterization of the Zhikong scallop (*Chlamys farreri*) mantle transcriptome and identification of biomineralization-related genes. Mar. Biotechnol..

[34] Li C., Weng S., Chen Y., Yu X., Lu L., Zhang H., He J., Xu X. (2012). Analysis of *Litopenaeus vannamei* transcriptome using the next-generation DNA sequencing technique. PLoS ONE.

[35] Wong Y.H., Ryu T., Seridi L., Ghosheh Y., Bougouffa S., Qian P.-Y., Ravasi T. (2014). Transcriptome analysis elucidates key developmental components of bryozoan lophophore development. Sci. Rep..

[36] Patnaik B.B., Hwang H.-J., Kang S.W., Park S.Y., Wang T.H., Park E.B., Chung J.M., Song D.K., Kim C., Kim S. (2015). Transcriptome characterization of non-model endangered lycaenids, *Protantigius superans* and *Spindasis takanosis*, using Illumina Hi-Seq 2500 sequencing. Int. J. Mol. Sci..

[37] Kang S.W., Park S.Y., Patnaik B.B., Hwang H.J., Kim C., Kim S., Lee J.S., Han Y.S., Lee Y.S. (2015). Construction of PANM Database (Protostome DB) for rapid annotation of NGS data in Mollusks. Korean J. Malacol..

[38] Mittapalli O., Bai X., Mamidala P., Rajarapu S.P., Bonello P., Herms D.A. (2010). Tissue specific transcriptomics of the exotic invasive insect pest Emerald Ash Borer (*Agrilus planipennis*). PLoS ONE.

[39] Liang S., Wang B., Pan L., Ye Y., He M., Han S., Zheng S., Wang X., Lin Y. (2012). Comprehensive structural annotation of *Pichia pastoris* transcriptome and the response to various carbon sources using deep-paired RNA sequencing. BMC Genomics.

[40] Simakov O., Marletaz F., Cho S.J., Edsinger-Gonzales E., Havlak P., Hellsten U., Kuo D.H., Larsson T., Lv J., Arendt D. (2013). Insights into bilateria evolution from three spiralian genomes. Nature.

[41] Heyland A., Vue Z., Voolstra C.R., Medina M., Moroz L.L. (2011). Developmental transcriptome of *Aplysia californica*. J. Exp. Zool. B Mol. Dev. Ecol..

[42] Consea A., Gotz S., Garcia-Gomez J.M., Terol J., Talon M., Robles M. (2005). Blast2go: A universal tool for annotation, visualization and analysis in functional genomics research. Bioinformatics.

[43] Gotz S., Garcia-Gomez J.M., Terol J., Williams T.D., Nagaraj S.H., Nueda M.J., Robles M., Talon M., Dopazo J., Conesa A. (2008). High-throughput functional annotation and data mining with the Blast2GO suite. Nucl. Acids Res..

[44] Du Plessis L., Skunca N., Dessimoz C. (2011). The what, where, how and why of gene ontology—A primer for bioinformaticians. Brief Bioinform..

[45] Lovering R.C., Camon E.B., Blake J.A., Diehl A.D. (2008). Access to immunology through the Gene Ontology. Immunology.

[46] Rhee S.Y., Wood V., Dolinski K., Draghici S. (2008). Use and misuse of the gene ontology annotations. Nat. Rev. Genet..

[47] Barell D., Dimmer E., Huntley R.P., Binns D., O’Donovan C., Apweiler R. (2009). The GOA database in 2009—An integrated Gene Ontology Annotation resource. Nucl. Acids Res..

[48] Ogata H., Goto S., Sato K., Fujibuchi W., Bono H., Kanehisa M. (1999). KEGG: Kyoto Encyclopaedia of Genes and Genomes. Nucl. Acids Res..

[49] Annadurai R.S., Neethiraj R., Jayakumar V., Damodaran A.C., Rao S.N., Katta M.A.V.S.K., Gopinathan S., Sarma S.P., Senthilkumar V., Niranjan V. (2013). De novo transcriptome assembly (NGS) of *Curcuma longa* L. rhizome reveals novel transcripts related to anticancer and antimalarial terpenoids. PLoS ONE.

[50] Lv J., Liu P., Gao B., Wang Y., Wang Z., Chen P., Li J. (2014). Transcriptome analysis of the *Portunus trituberculatus*: De novo assembly, growth related gene identification and marker discovery. PLoS ONE.

[51] Ma H., Ma C., Li S., Jiang W., Li X., Liu Y., Ma L. (2014). Transcriptome analysis of the Mud Crab (*Scylla paramamosain*) by 454 deep sequencing: Assembly, annotation and marker discovery. PLoS ONE.

[52] Khush R.S., Leulier F., Lemaitre B. (2001). *Drosophila* immunity: Two paths to NF-ĸB. Trends Immunol..

[53] Brayer K.J., Segal D.J. (2008). Keep your fingers off my DNA: Protein-protein interactions mediated by C2H2 zinc finger domains. Cell Biochem. Biophys..

[54] Pairett A.N., Serb J.M. (2013). De novo assembly and characterization of two transcriptomes reveal multiple light-mediated functions in the Scallop eye (Bivalvia: Pectinidae). PLoS ONE.

[55] Albertin C.B., Simakov O., Mitros T., Wang Z.Y., Pungor J.R., Edsinger-Gonzalez E., Brenner S., Ragsdale C.W., Rokhsar D.S. (2015). The octopus genome and the evolution of cephalopod neural and morphological novelties. Nature.

[56] Wang L., Wang L., Huang M., Zhang H., Song L. (2011). The immune role of C-type lectins in molluscs. ISJ.

[57] Wang X., Li J., Li Y. (2015). Isolation and characterization of Microsatellite markers for an endemic tree in East Asia, *Quercus variabilis* (Fagaceae). Appl. Plant Sci..

[58] Frankham R. (2003). Genetics and conservation biology. Comptes Rendus.

