# Supplementary material for: Transcriptomic Analysis of the Endangered Neritid Species Clithon retropictus: De Novo Assembly, Functional Annotation, and Marker Discovery
Source: Genes (Basel). 2016 Jul 22;7(7):35. doi: 10.3390/genes7070035 (PMC4962005; doi:10.3390/genes7070035)
Supplement: Supplementary file 1 [file genes-07-00035-s001.zip › genes-131355-supplementary/genes-131355-Table S1.docx]

**Supplementary Materials: Transcriptomic Analysis of the Endangered Neritid Species *Clithon retropictus*: *De Novo* Assembly, Functional Annotation, and Marker Discovery**

So Young Park, Bharat Bhusan Patnaik, Se Won Kang, Hee-Ju Hwang, Jong Min Chung,
Dae Kwon Song, Min Kyu Sang, Hongray Howrelia Patnaik, Jae Bong Lee, Mi Young Noh, Changmu Kim, Soonok Kim, Hong Seog Park, Jun Sang Lee, Yeon Soo Han and Yong Seok Lee

**Table S1.** Pre-processing of raw reads using the Cutadapt program.

| Total number of raw reads | |
| --- | --- |
| Number of sequences | 246,743,798 |
| Number of bases | 31,089,718,548 |
| Total read pairs processed | 123,371,899 |
| Read 1 with adapter | 4,663,494 (3.8%) |
| Read 2 with adapter | 5,066,850 (4.1%) |
| Pairs written (passing filters) | 123,371,899 |
| Total base pairs processed (bp) | 31,089,718,548 |
| Read 1 (bp) | 15,544,859,274 |
| Read 2 (bp) | 15,544,859,274 |
| Total written (filtered) (bp) | 31,030,569,174 |
| Read 1 (bp) | 15,515,894,689 |
| Read 2 (bp) | 15,514,674,485 |
| Average length after trimming (bp) | 125.8 |
| Percent of reads Discarded (%) | 0.2% |

| Adapter 1 sequence | AGATCGGAAGAGCACACGTCTGAACTCCAGTCAC |
| --- | --- |
| Adapter 2 sequence | AGATCGGAAGAGCGTCGTGTAGGGAAAGAGTGTAGATCTCGGTGGTCGCCGTATCATT |

© 2016 by the authors. Submitted for possible open access publication under
the terms and conditions of the Creative Commons Attribution (CC-BY) license (<http://creativecommons.org/licenses/by/4.0/>).
